# Radiation driven epithelial-mesenchymal transition is mediated by Notch signaling in breast cancer

**DOI:** 10.18632/oncotarget.10802

**Published:** 2016-07-23

**Authors:** Rae-Kwon Kim, Neha Kaushik, Yongjoon Suh, Ki-Chun Yoo, Yan-Hong Cui, Min-Jung Kim, Hae-June Lee, In-Gyu Kim, Su-Jae Lee

**Affiliations:** ^1^ Department of Life Science, College of Natural Sciences, Hanyang University, Seoul, Korea; ^2^ Laboratory of Radiation Exposure and Therapeutics, National Radiation Emergency Medical Center, Korea Institute of Radiological and Medical Sciences, Seoul, Korea; ^3^ Division of Radiation Effect, Korea Institute of Radiological and Medical Sciences, Seoul, Korea; ^4^ Department of Radiation Biology, Environmental Radiation Research Group, Korea Atomic Energy Research Institute, Daejeon, Korea

**Keywords:** radiation, EMT, Notch signaling, interleukin-6, breast cancer

## Abstract

Epithelial to mesenchymal transition (EMT) is developmental process associated with cancer metastasis. Here, we found that breast carcinoma cells adopt epithelial-to-mesenchymal transition (EMT) in response to fractionated-radiation. Importantly, we show that Notch signaling is highly activated in fractionally-irradiated tumors as compared to non-irradiated tumors that are accompanied by an EMT. Moreover, we uncovered the mechanism of Notch-driven EMT, in which Notch enhanced EMT through IL-6/JAK/STAT3 signaling axis in mammary tumor cells. Collectively, we present converging evidence from our studies that Notch2 is a critical mediator of radiation-induced EMT and responsible for induced malignant tumor growth.

## INTRODUCTION

Most cancer deaths from breast cancer result from tumor recurrence following treatment of the primary tumor. Ionizing radiation (IR) is the preferred treatment strategy for curing cancers at advanced stages including breast cancer. However, the emergence of radio-resistance leads to the failure of radiotherapy and subsequently increases mortality frequently in patients [1]. These consequences suggest that a subcellular fraction of radiation resistant tumor cells with potent tumorigenic activity is critical for re-growth [2–4]. To overcome these problems, determining the underlying molecular mechanisms associated with radiation-induced epithelial-to-mesenchymal transition (EMT) will be helpful for breast cancer relapse predictions and would greatly improve the therapeutic approaches for this disease [5–7]. Undertaking this aim, we focused on epithelial-to-mesenchymal transition (EMT) phenomenon in breast tumor cells.

The process of EMT is crucial in metastatic dissemination and has been the subject of intense investigations [8]. EMT is controlled by multifaceted pathways in which differentiated epithelial cells undergo morphological modifications from an epithelial cobblestone-like type to an elongated mesenchymal fibroblast type including decreased expression in epithelial markers such as E-cadherin with increased expression of mesenchymal markers such as N-cadherin and vimentin [9, 10]. It is widely accepted that EMT plays a key role in the radio-resistance phenomenon and has been broadly explored in several types of tumors such as gliomas and breast, and lung cancer [11–13]. Recently, it is well-believed that EMT is also triggered by extracellular stimuli such as hypoxia and radiation [14].

Accumulating evidences suggests that Notch signaling involved in many physiological processes including call fate control during neurogenesis, cancer stem cell maintenance, EMT, and differentiation [15–17]. Notch signaling can be activated through four transmembrane Notch receptors (Notch 1–4) that can bind with Notch family ligands (Jagged-1, Jagged-2, Delta-like 1, 3, and 4) [18]. Following ligand binding, Notch receptors undergo a series of proteolytic cleavages and the released Notch intracellular domain (NICD) translocate to the nucleus where it interacts with the DNA binding protein CSL (C protein binding factor 1/Suppressor of Hairless/Lag-1), transforming it from a transcriptional repressor to an activator by recruiting cofactors. However, the role of Notch signaling in radiation-induced EMT remains largely unknown.

Independent of these findings, some cytokines, secreted by cells within tumor microenvironment, is critical to stimulate EMT [19]. Among all of these, IL-6 can promote tumorigenicity, angiogenesis, and metastasis [20, 21]. In addition, IL-6 has been shown to be a direct regulator of the self-renewal of breast cancer stem cells as mediated by the IL-6 receptor/GP130 complex through STAT3 activation [22]. An elevated level of IL-6 mediated Jagged1/Notch signaling was also proposed to promote breast cancer bone metastasis [23]. These studies suggest a functional role of IL-6 with regard to malignant progression and metastasis. Still, mechanisms of contributory role of IL-6 in facilitating the progression from a pre-malignant state to a state of metastasis remain obscure.

The challenge to establish how fractionated radiation contributes to the EMT acquiring acquisition of malignant phenotypes following exposure doses in cancer patients remains unexplored. In this study, we investigated that Notch signaling is critical for fractionated radiation-induced EMT. Mechanistically, we uncover an innovative mechanism of the regulation of Notch2 via the JAK/STAT3 signaling pathway and subsequently-induced tumor malignancy in response to the radiation. Furthermore, we find that radiation-induced IL-6 secretion serves as a major contributor to Notch-induced EMT in breast cancer cells in response to the fractionated radiation.

## RESULTS

### Radiation promotes the induction of mesenchymal traits associated with EMT in breast cancer cells

**Figure 1:**
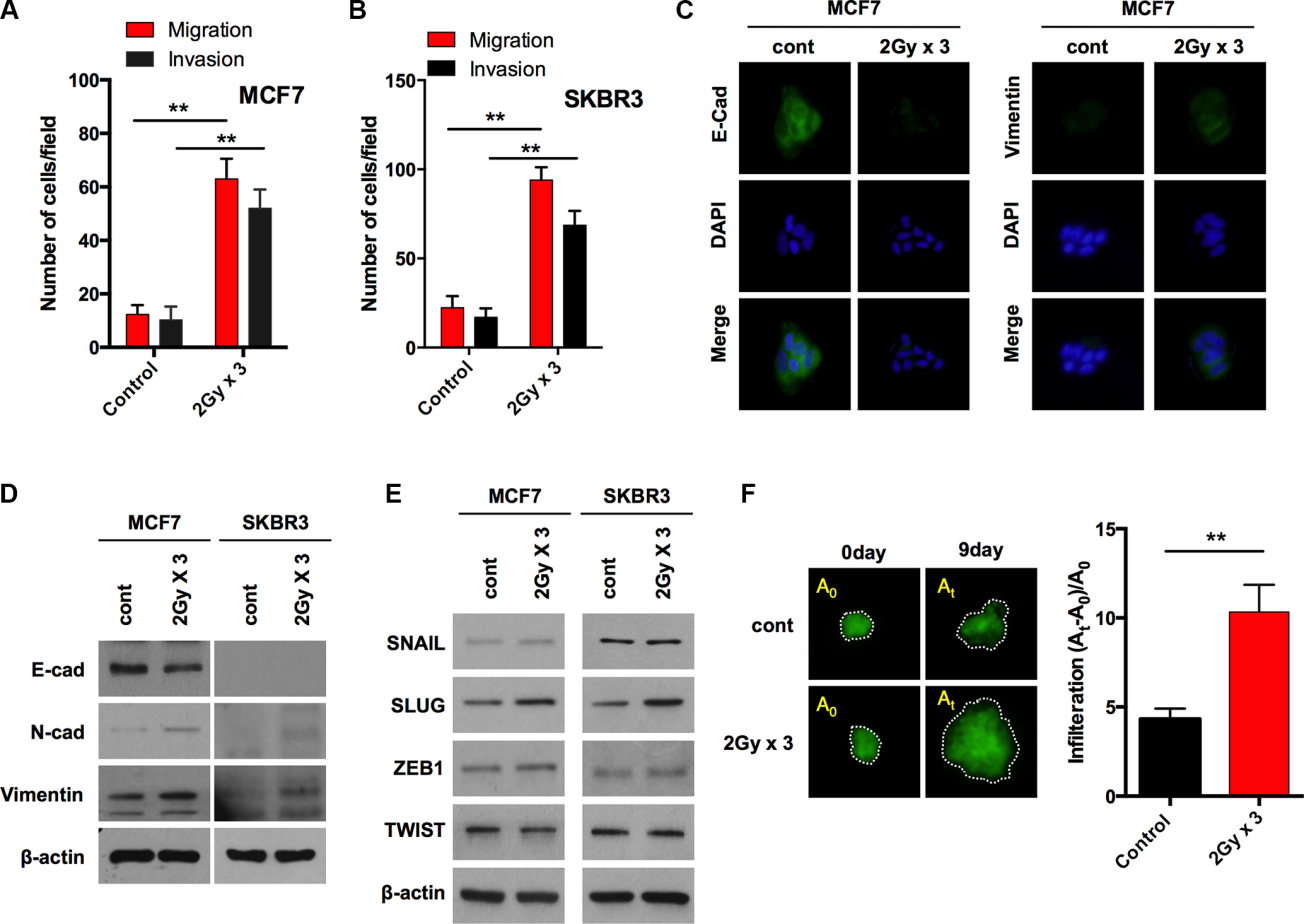
Fractionated radiation promotes invasiveness of breast cancer cells through EMT (**A** and **B**) Migration and invasion assay of MCF7 and SKBR3 breast cancer cells in transwells after fractionated irradiation (2 Gy × 3), respectively. (**C**) Immunocytochemistry for EMT markers such as E-cadherin and Vimentin in MCF7 cancer cells after irradiation. (**D**) Protein expression of EMT markers such as E-cadherin, N-cadherin and Vimentin in MCF7 and SKBR3 breast cancer cells after irradiation (**E**) Protein expression of EMT transcription factors such as SNAIL, SLUG, ZEB1 and TWIST in MCF7 and SKBR3 breast cancer cells after irradiation. (**F**) Invasion of GFP-transduced MCF7 spheroid cells treated in collagen-based matrix 3D culture system following irradiation. A_0_ represents the area of sphere at day 0 while At is the area at day 9. β-actin was used as a loading control. Error bars represent mean ± S.D. of triplicate samples. *p < 0.05, and **p < 0.01.

To explore the possible harmful effect of IR, we tested whether IR causes breast cancer cells to acquire mesenchymal traits. Elevated cell migration is one of the characteristic features of cells undergoing EMT. Accordingly, we initially examined the migration and invasion in MCF7 and SKBR3 cells. To this end, both cancer cells were exposed to fractionated doses of radiation (2 Gy × 3; 2 Gy for consistent 3 days). Notably, migration and invasion were significantly increased in both cells after fractionated radiation (Figure 1A and 1B). However, the cell growth rate was significantly decreased by the fractionated radiation in both cell lines with post incubation time (Supplementary Figure S1A), suggesting that radiation is able to induce EMT while also inhibiting cell growth. Loss of E-cadherin and an increase in vimentin exhibited morphological modifications in MCF7 cells as compared to un-irradiated cells after fractionated radiation (Figure 1C). In addition, E-cadherin protein levels were downregulated and N-cadherin and vimentin protein levels were up-regulated in response to the fractionated radiation in MCF7 cells (Figure 1D). Concomitantly, when the protein levels of EMT transcription factors such as SNAIL, SLUG, ZEB1 and TWIST were investigated, irradiation was found to cause a strong increase of SLUG; however, other EMT transcription factors were not altered by irradiation in MCF7 breast cancer cells (Figure 1E). To confirm this phenomenon, the invasiveness of fractionated-radiation exposed MCF7 cells was visualized and quantified into a collagen-based matrix. Consistent with the results of Transwell boyden chamber invasion assays, the invasiveness was highly enhanced in MCF7 cells after fractionated radiation at day 9 (Figure 1F). To test the involvement of SLUG in radiation-induced EMT further, MCF7 breast cancer cells were transfected with small interfering RNA (siRNA) targeting SLUG prior to a radiation treatment. Of note, the knock-down of SLUG effectively decreased the radiation-induced migratory and invasive properties of both MCF7 and SKBR3 breast cancer cells (Supplementary Figure S1B and S1B). Importantly, the downregulation of E-cadherin protein levels was retained and the accumulation of N-cadherin and vimentin was abolished with the knockdown of SLUG (Supplementary Figure S1D) in MCF7 cells treated with fractionated radiation. In addition, immunostaining data revealed that E-cadherin was further restored while N-cadherin and vimentin levels were diminished in radiation treated MCF7 cells (Supplementary Figure S1E). Taken together, these results suggest that radiation causes breast cancer cells to acquire migratory and invasive properties by inducing SLUG and thereby triggering the EMT program.

### Radiation triggers EMT through the activation of Notch signaling

**Figure 2:**
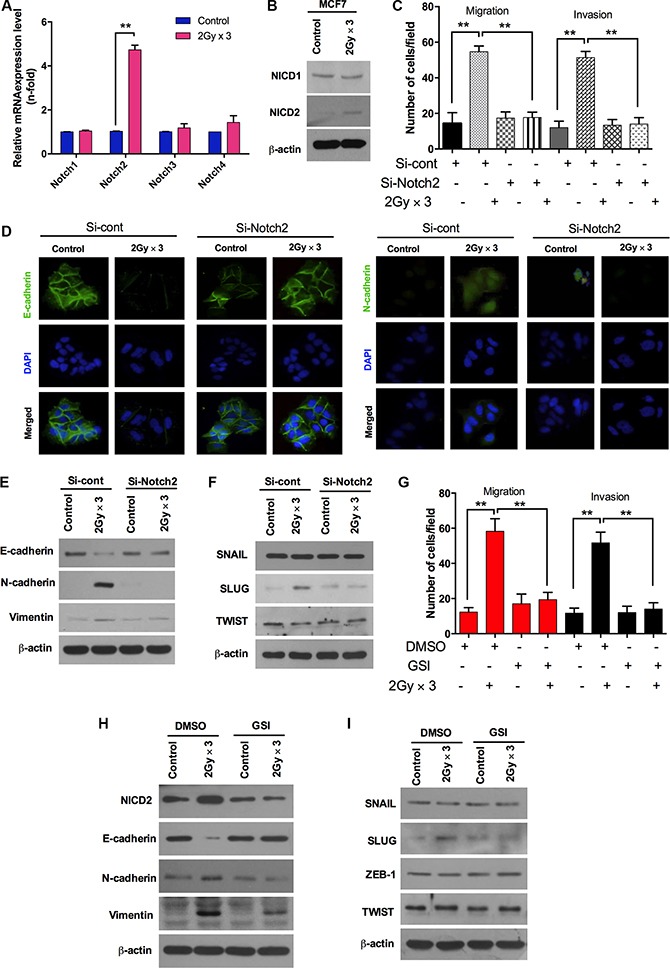
Fractionated radiation-induced Notch signaling promotes EMT in breast cancer cells (**A**) qRT PCR analysis for mRNA levels of Notch family members (Notch1-4) in MCF7 breast cancer cell after fractionated irradiation. (**B**) Protein expression of Notch family such as Notch1 (NICD1) and Notch2 (NICD2) in MCF7 after fractionated irradiation. (**C**)) Migration and invasion assay in transwells after irradiation of MCF7 that are transfected with siRNA targeting Notch2. (**D**) Immunocytochemistry for EMT markers (E-cadherin and N-cadherin) after fractionated irradiation of MCF7 that are treated with siRNA targeting Notch2. (**E**) Western blot for EMT markers such as E-cadherin, N-cadherin and Vimentin in MCF7 cells that are transfected with siRNA targeting Notch2 prior to irradiation. (**F**) Western blots for EMT transcription factors such as SNAIL, SLUG and TWIST in MCF7 cells that are transfected with siRNA targeting Notch2 and irradiated afterwards. (**G**) Migration and invasion assay in transwells after irradiation of MCF7 that are treated with gamma-secretase inhibitor, well known Notch signaling inhibitor (GSI, 20 μM). (**H** and **I**) Western blot for EMT markers and regulators after fractionated irradiation in MCF7 cells that are treated with pretreated with GSI. β-actin was used as a loading control. Error bars represent mean ± S.D. of triplicate samples. *p < 0.05, and **p < 0.01.

Recently, the Notch signaling pathway has widely accepted as a vital regulator during the induction of EMT. It has been suggested as a direct target of SLUG via regulating the SLUG promoter through the CSL interaction, resulting in the up-regulation of SLUG in endothelial cells [24–26]. In line with these studies, to understand the invasiveness of cells in response to the fractionated radiation, we subsequently examined the mRNA expression levels of the Notch family. Interestingly, Notch2 mRNA levels were markedly increased in MCF7 cells after radiation, while other Notch family components such as Notch1, Notch3 and Notch4 remained unchanged (Figure 2A). Also, the activation of Notch2 (NICD2) is noticeably increased through cleavage followed by fractionated radiation (Figure 2B). In addition, to extend our observations, MCF7 cells were transfected with siRNA targeting Notch2. Down-regulation of Notch2 significantly blocked migration and invasion in MCF7 cells in response to radiation treatment (Figure 2C). Moreover, immunostaining data revealed that E-cadherin was restored and the N-cadherin was abolished with the knock-down of Notch2 in radiation-treated MCF7 cells (Figure 2D). Accordingly, protein levels of E-cadherin were up-regulated and accumulation of N-cadherin and vimentin was abolished after knock-down of Notch2 in irradiated MCF7 breast cancer cells (Figure 2E). It is worth mentioning here that the knock-down of Notch2 effectively down-regulates SLUG protein levels in irradiated MCF7 cells; however other transcription factors such as SNAIL and TWIST remain unchanged (Figure 2F). Furthermore, the down-regulation of Notch2 decreased the clonogenic survival rate in irradiated MCF7 and SKBR3 cells (Supplementary Figure S2A and S2B). To confirm the involvement of Notch in fractionated radiation-induced EMT in breast cancer cells, we blocked Notch signaling using a gamma secretase inhibitor (GSI). The inhibition of Notch2 activation attenuated the radiation-induced invasive and migratory properties of MCF7 breast cancer cells (Figure 2G) and recovered the E-cadherin protein levels with the down-regulation of N-cadherin, vimentin and NICD2 (Figure 2H). These changes also mitigate radiation-induced SLUG induction in irradiated MCF7 cells (Figure 2I). Collectively, these findings suggest that Notch2 regulates radiation-induced EMT responses via the NICD2 domain in breast cancer cells.

### The expression of Notch ligands is required for radiation-induced Notch activation leading to EMT

**Figure 3:**
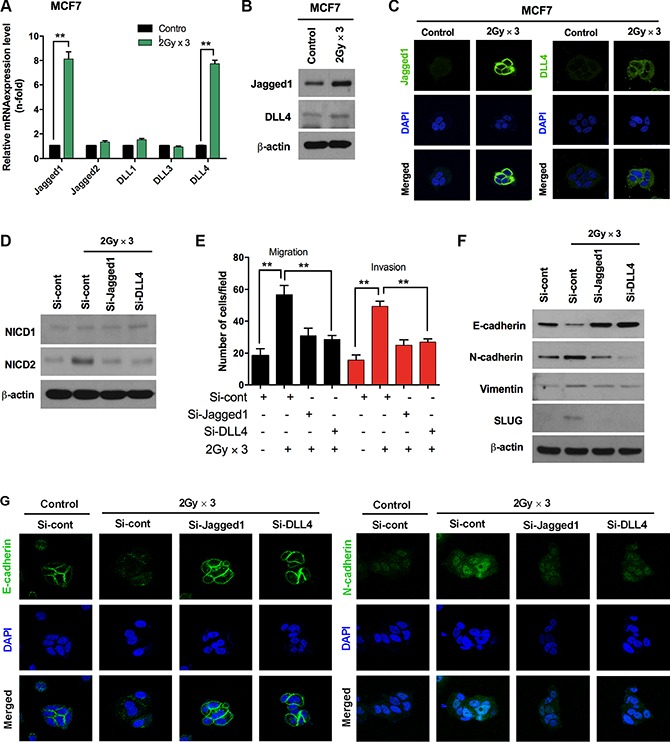
Fractionated radiation-induced Notch ligands promote EMT in breast cancer cells (**A**) qRT PCR analysis for mRNA levels of Notch ligands in MCF7 breast cancer cell after fractionated irradiation. (**B**) Western blot and (**C**) immunocytochemistry for Notch ligands such as Jagged1 and DLL4 in MCF7 breast cancer cells after irradiation. (**D**) Protein expression for Notch2 activation in irradiated MCF7 cells that are transfected with siRNA targeting Jagged1 and DLL4. (**E**) Migration and invasion assays in transwells after irradiation of MCF7 that are transfected with siRNA targeting Jagged1 and DLL4. (**F**) Western blot and (**G**) immunocytochemistry for EMT markers after fractionated irradiation of MCF7 cells transfected with siRNA targeting Jagged1 and DLL4. β-actin was used as a loading control. Error bars represent mean ± S.D. of triplicate samples. *p < 0.05, **p < 0.01 and ***p < 0.001.

As the Jagged-1 mediated activation of Notch signaling is important during the induction of EMT [27], we subsequently sought to determine whether Notch ligands are expressed in breast cancer cells due to fractionated radiation. Since the Notch2 receptor is recognized in irradiated breast cancer cells and the intracellular signaling is transduced by Notch ligands. By analyzing multiple Notch ligands, we found that Jagged1 and DLL4 mRNA were increased significantly in irradiated MCF7 cells as compared to un-irradiated cells, whereas the smaller changes noted in Jagged2, and DLL1 transcripts did not reach statistical significance (Figure 3A). Additionally, elevated Jagged1 and DLL4 mRNA levels resulted in increased accumulation of protein levels in irradiated MCF7 cells as compared with un-irradiated cells (Figure 3B). Immunocytochemistry also exposed higher expression levels of Jagged1 and DLL4 in MCF7 cells after fractionated radiation (Figure 3C). Due to the strong accumulation of Jagged1 and DLL4 in response to radiation, we decided to analyze the effects of Jagged1 and DLL4 inhibition in irradiated MCF7. Here we observed that NICD2 was significantly down-regulated after the knock-down of these ligands; however, NICD1 remained unchanged in irradiated MCF7 cells (Figure 3D). Interestingly, the knock-down of Jagged1 and DLL4 also blocked Notch-induced invasion and migration along with increased E-cadherin and decreased N-cadherin and vimentin protein levels in irradiated MCF7 cancer cells (Figure 3E and 3F). Restoration of pericellular E-cadherin together with attenuation of N-cadherin indicates that the expressions of Jagged1 and DLL4 are crucial for radiation activated Notch signaling for the induction of EMT (Figure 3G).

### Radiation upregulates Notch induced EMT through the activation of STAT3 signaling

**Figure 4:**
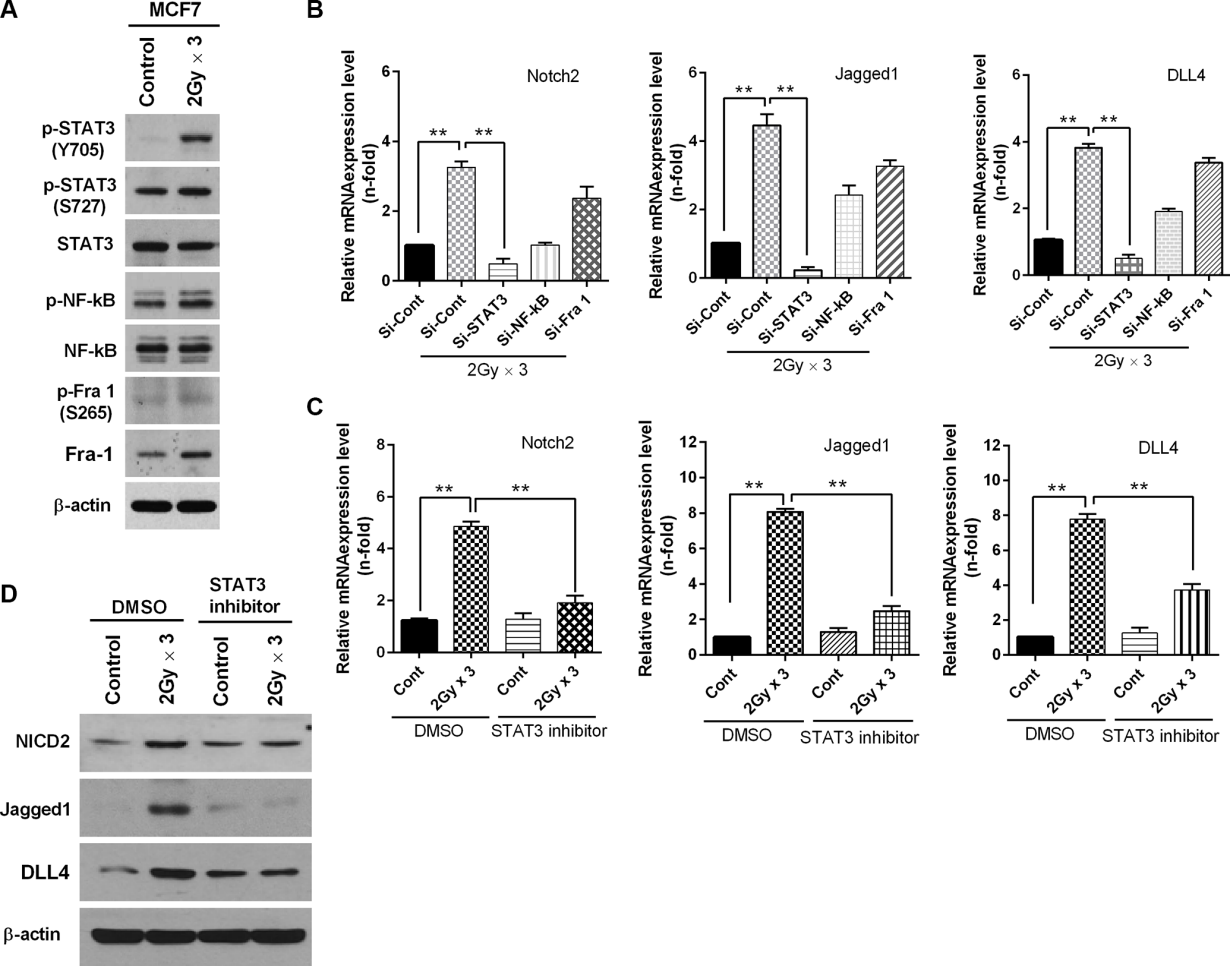
Fractionated radiation up-regulates Notch signaling through activation of STAT3 (**A**) Western blot analysis for STAT3, NF-κB and Fra-1 phosphorylation status rafter irradiation of MCF7 breast cancer cells. (**B**) qRT-PCR for Notch2, Jagged-1 and DLL4 mRNA levels after irradiation of MCF7 cells that are transfected with siRNA targeting STAT3, NF-κB and Fra-1. (**C**) qRT-PCR for Notch2, Jagged1 and DLL4 mRNA levels after irradiation of MCF7 cells that are treated with STAT3 inhibitor (2 μM). (**D**) Western blot for NICD2, Jagged1 and DLL4 after fractionated irradiation (2 Gy × 3) of MCF7 that are treated with STAT3 inhibitor (2 μM). Error bars represent mean ± S.D. of triplicate samples. *p < 0.05, **p < 0.01 and ***p < 0.001.

To gain further insight into the molecular mechanisms affecting the regulation of EMT, we sought to identify transcription factors whose expression regulates EMT through the radiation-induced Notch signaling pathway in breast cancer cells. To this end, first we examined the expression levels of the transcription factors such as STAT3, NF-κB and Fra-1 that are frequently associated with EMT signaling in breast cancer cells after fractionated irradiation. Notably, the activation of STAT3 was significantly induced by fractionated radiation in MCF7 cells, whereas the activation levels of NF-κB and Fra-1 remained unchanged (Figure 4A). In agreement with these results, the knock-down of STAT3 effectively attenuated radiation-induced Notch signaling (Figure 4B, Supplementary Figure S3A). Given that irradiation promoted the activation of STAT3, next we investigated whether STAT3 has significance in radiation-induced Notch2 activation. To this end, when MCF7 breast cancer cells were treated with a STAT3 inhibitor or DMSO and radiated afterwards, we observed that the inactivation of STAT3 sharply attenuated the radiation-induced Notch2 (NICD2), Jagged1 and DLL4 mRNA expression and protein levels (Figure 4C and 4D). Consistent with these results, interestingly the down-regulation of STAT3 also decreased the invasiveness and motility (Supplementary Figure S3B) and the expression of the epithelial marker such as E-cadherin was reappeared while the mesenchymal markers such as N-cadherin and vimentin were lost in irradiated MCF7 breast cancer cells (Supplementary Figure S3C and S3D). Because JAK (Janus kinases) is well-known regulator of STAT, we were curious to know whether the radiation-induced activation of STAT3 regulated by JAK could induce EMT through the Notch signaling pathway. Treatment with a JAK inhibitor effectively abrogates the radiation-induced invasive and migratory properties in irradiated MCF7 cells (Supplementary Figure S3E). In parallel, the inhibition of JAK mitigated the protein levels of N-cadherin and vimentin, as well as protein levels of SLUG, as induced by fractionated radiation (Supplementary Figure S3F).

### Radiation-induced IL-6 secretion mediates Notch signaling through the activation of STAT3 signaling

**Figure 5:**
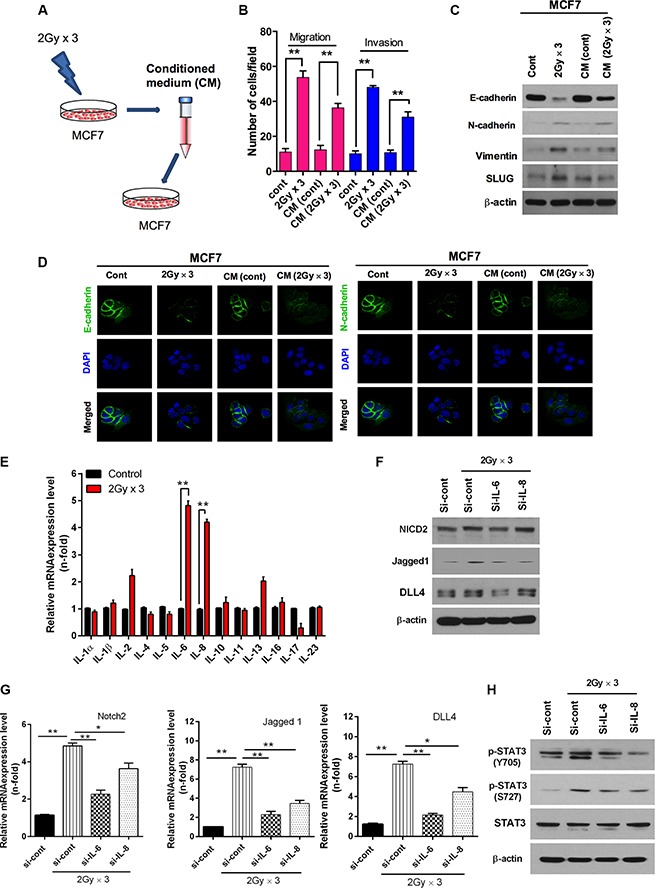
Radiation-induced IL6 secretion mediates activation of Notch signaling (**A**) Schematic model illustrating conditioned medium treated MCF7 breast cancer cells. MCF7 cells were irradiated 2 Gy × 3 as mentioned earlier. Then cell's medium was further harvested and treated to another MCF7 cells. (**B**) Migration and invasion assay in transwells after irradiated cultured medium those are compared with directly irradiated MCF7 cells. (**C**) Western blot and (**D**) immunocytochemistry for EMT markers after fractionated irradiation of MCF7 that are treated with irradiated cultured medium. (**E**) qRT PCR analysis for mRNA levels of cytokines family in MCF7 cells after fractionated irradiation. (**F**) Western blot for NICD2, Jagged1 and DLL4 after fractionated irradiation of MCF7 that are transfected with siRNA targeting IL-6 and IL-8. (**G**) qRT-PCR for Notch2, Jagged-1 and DLL4 mRNA levels after irradiation of MCF7 cells that are transfected with siRNA targeting IL-6 and IL-8. (**H**) Western blot for activation of STAT3 after fractionated irradiation of MCF7 that are transfected with siRNA targeting IL-6 and IL-8. β-actin was used as a loading control. Error bars represent mean ± S.D. of triplicate samples. *p < 0.05 and **p < 0.01.

Because the signal transduction of IL-6 is involved in the activation of JAK leading to the activation of transcription factor STAT3 [28], we questioned if a certain secretion factor secreted by radiation could induce EMT. To this end, MCF7 breast cancer cells were irradiated at 2 Gy × 3 as previously described and other MCF7 cells were treated with a conditioned medium that has a secretion factors induced by MCF7 after irradiation (Figure 5A). Both migration and invasion levels were significantly increased in both treated conditions in response to radiation (Figure 5B). Remarkably, the expression levels of mesenchymal marker proteins were increased after treatment with radiation irradiated culture medium in MCF7 cells (Figure 5C). Loss of E-cadherin again confirms that some secretion factors were induced in culture medium by MCF7 cells in response of radiation (Figure 5D). Moreover, by analyzing multiple ILs, we found that the expression levels of IL-6 and IL-8 transcripts were markedly increased in MCF7 cells by radiation among all cytokines tested (Figure 5E). Owing to the robust induction of IL-6 and IL-8 in irradiated MCF7 cells, we sought to determine if IL-6 and IL-8 play significant roles in radiation-induced Notch2 signaling expression. As expected, the knock-down of IL-6 decreased the radiation-induced Notch2, Jagged1 and DLL4 protein levels; however, IL-8 partially inhibited Notch2, Jagged1 and DLL4 protein levels (Figure 5F). Indeed, Jagged1, DLL4 and Notch2 transcripts were significantly down-regulated by the IL-6 and IL-8 knock-down in irradiated MCF7 cells (Figure 5G). In contrast, EMT was induced more effectively by recombinant IL-6 protein than IL-8 in MCF7 cells (Supplementary Figure S4). Meanwhile, the activation of STAT3 did not show increase due to fractionated radiation by the knock-down of IL-6 and IL-8 in irradiated MCF7 breast cancer cells (Figure 5H). Next, to confirm the role of IL-6 signaling in radiation-induced EMT, its activity was inhibited in irradiated MCF7 cells by a neutralizing antibody. As expected, treatment with the IL-6 neutralizing antibody effectively decreased radiation-induced invasion and migration in MCF7 cells (Supplementary Figure S5A). Consistent with these results, the expression of the epithelial marker E-cadherin was also increased and the mesenchymal markers N-cadherin and vimentin were decreased by fractionated radiation after treatment with the IL-6 neutralizing antibody in MCF7 cells. Remarkably, Slug protein levels were also down-regulated by the IL-6 neutralizing antibody in MCF7 cells in response to radiation (Supplementary Figure S5B). Immunostaining analyses of the increased E-cadherin and diminished N-cadherin levels confirm that IL-6 upregulates radiation-induced EMT responses in cancer cells (Supplementary Figure S5C). These observations taken together suggest that IL-6 strongly mediates Notch-induced EMT through the JAK/STAT3 signaling axis in breast cancer cells.

### Radiation-induced Notch promotes EMT in mice

**Figure 6:**
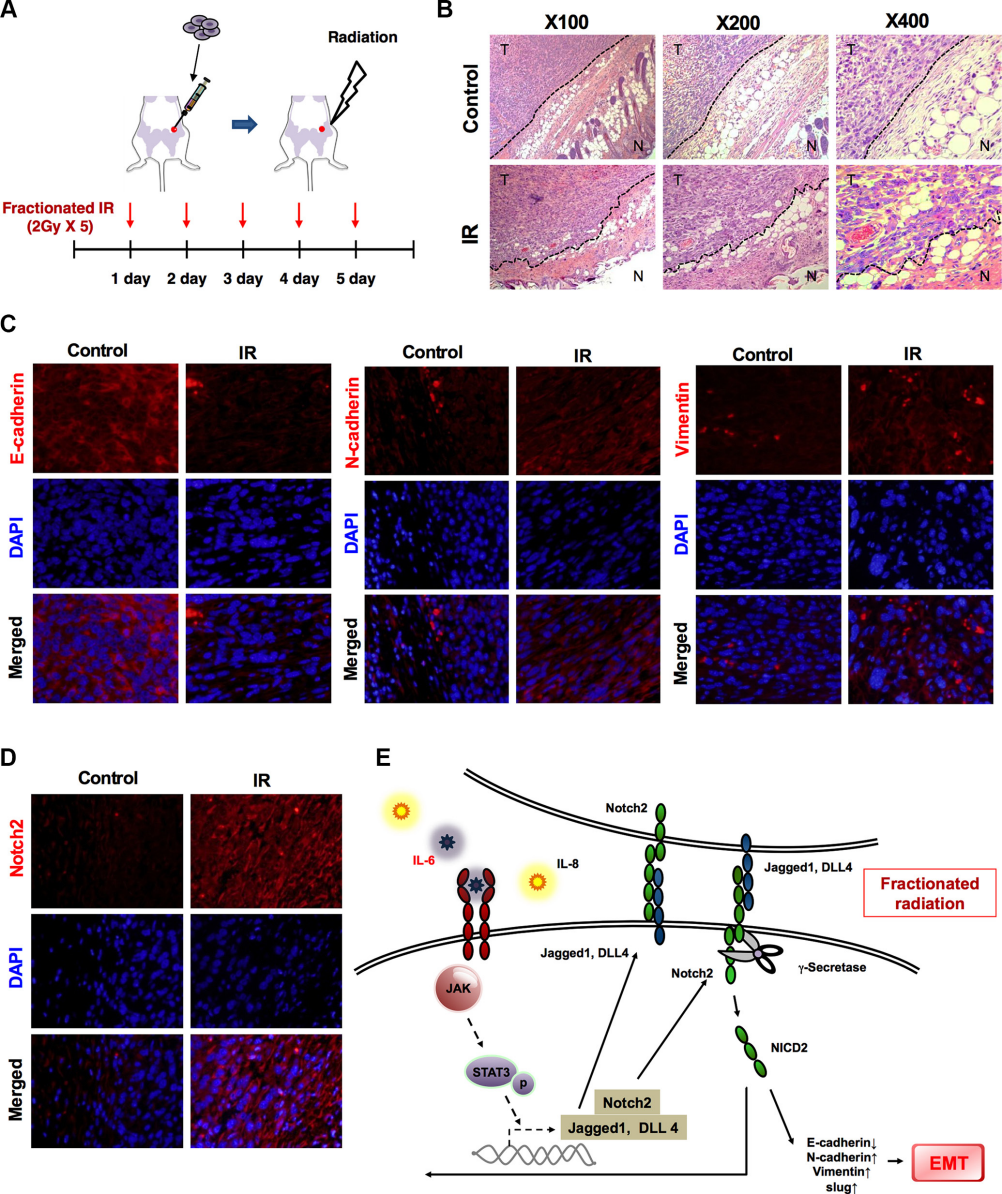
Radiation-induced Notch promotes EMT in mice (**A**) Schematic representation of experimental scheme for treating *in vivo* mice model by fractionated radiation (2 Gy/day for five consistent days). (**B**) Hematoxylin and eosin (H&E) staining of breast tumor tissue in mice 24 hour after irradiation as described above. (**C**) Immunohistochemistry for EMT markers such as E-cadherin, N-cadherin and vimentin in breast tumor tissues of mice after irradiation. (**D**) Immunohistochemistry for expression of Notch2 in breast cancer tissues of mice after irradiation with 10 Gy (2 Gy × 5). (**E**) Schematic model that fractionated radiation promotes EMT through Notch signaling in breast cancer cells. Importantly, irradiation-activated IL-6 increases the Notch-activity via activating JAK/ STAT3 for EMT.

The epithelial to mesenchymal transformation, along with the increased invasiveness and motility of breast cancer cells *in vitro*, prompted us to analyze the effects of fractionated radiation (2 Gy × 5) *in vivo* (Figure 6A). Two-three days after an orthotropic injection of 4T1 breast tumor cells in mice, irradiation was performed. Histological staining indicated that tumor cells were disseminating from their margins in irradiated tissues as compared to non-irradiated cases (Figure 6B). These findings indicated that cancer cells gain migratory properties and going to metastasize after radiation. *In vitro* data also revealed that 4T1 mouse breast tumor cells showed higher rate of migration and invasion along with decreased E–cadherin and increased N-cadherin and vimentin levels after fractionated radiation (Supplementary Figure S6A and S6B). Additionally, noticeable losses of E-cadherin expression levels and increased N-cadherin, vimentin levels were detected in irradiated tumors (Figure 6C). Remarkably, Notch2 was also overexpressed in irradiated tumors compared to non-irradiated cases (Figure 6D). Therefore, we conclude that radiation-induced Notch activation can increase the EMT in breast cancer cells *in vivo*.

## DISCUSSION

Recently EMT has been considered to have malignant features in tumor cells, causing these cells to gain a tendency towards metastatic dissemination, resistance to various chemotherapies, and relapse. Several reports now stated that radiation is commonly considered as one of the well-known inducer of EMT [29–31]. Hence, understanding the molecular mechanisms acting with regard to radiation towards EMT is necessary to generate novel therapeutic procedures.

In the present study, we identified Notch signaling as a critical regulator of mammary tumor progression by ionizing radiation. Notch expression is significantly up-regulated in irradiated tumors arising in mice bearing mammary tumors relative to non-irradiated tumors. Interestingly, Notch2 induction accelerated tumor malignancy along with increased mesenchymal markers, indicating that Notch2 is necessary and sufficient for the loss of E-cadherin and increased N-cadherin and vimentin levels. Finally, we defined the molecular basis of radiation-induced EMT in breast cancer cells, finding that it results from the fact that Notch2 upregulation is accompanied by IL-6-dependent STAT3 activation. These findings provide a mechanistic link between Notch2 induction and IL-6 secretion for the progression of tumor malignancy in mammary tumor cells.

Importantly, our findings reveal that Notch2 inhibition effectively altered the expression of mesenchymal markers that requires SLUG activity. The SNAIL family members including SLUG encode zinc finger–containing transcriptional activators that promote EMT during tumor development, in part by regulating the expression of E-cadherin junctional proteins [32]. In line with this notion, a recent report demonstrates that Notch controls expression of SLUG and that abrogation of Notch signaling prevents tumor growth and metastasis in an *in vivo* tumor model [33]. In contrast to previous studies, we find here that the transcriptional activator SLUG, but not SNAIL, participates in the Notch-activated EMT pathway [26]. Remarkably, SLUG knockdown restored E-cadherin expression in breast cancer cells while also decreasing mesenchymal markers in response to radiation. Consistent with this, we observed that a γ-secretase inhibitor treatment reversed these pro-metastatic functions of Notch2 by disrupting the Notch pathway in associated breast cancer cells [34]. These findings broaden our understanding of radiation regulated EMT processes through Notch pathways and open possible new avenues for cancer therapeutics improvement via inhibition of Notch-mediated tumor metastasis.

We also demonstrated that elevated levels of IL-6 can be detected in the breast cancer cells after radiation. Higher levels of IL-6 have a generally been reported to be correlated with a more advanced stage of disease and a worse outcome [35]. Of note, we observed that fractionated radiation increased the secretion of IL-6 in the tumor microenvironment through the activation of the JAK/STAT3/Notch2 signaling pathway as shown in a schematic model (Figure 6E). Collectively, our findings suggest that Notch2 could be a therapeutic target of efforts to overcome radiotherapy induced relapse in the breast cancer treatment. Hence, treatment of Notch inhibitors, prior to radiotherapy, in tumors at risk for metastasizing may be a fruitful approach in the direction of improving cancer therapeutic outcomes.

## MATERIALS AND METHODS

### Chemical reagents and antibodies

Monoclonal antibodies to N-cadherin (610920) and E-cadherin (612130) were purchased from BD Transduction Laboratory (Seoul, Korea). Polyclonal antibodies to STAT3 (sc-482), NF-κB (p65) (sc- 109), Vimentin (sc-5565), SLUG (sc-10436) and TWIST (sc- 15393) were purchased from Santa Cruz Biotechnology (Santa Cruz, CA, USA). The polyclonal antibody to ZEB1 (HPA027524) was purchased from Sigma (St Louis, MO, USA). The polyclonal antibodies to Snail (3879), p-STAT3 (Y705) (9131), p-STAT3 (S727) (9134), Fra1 (5281), p-Fra1 (3880) and Notch1 (2421) were obtained from Cell Signaling Technology (Beverly, MA, USA). Polyclonal antibodies to anti-IL-6 (ab6672), Notch2 (ab72803), Jagged1 (ab109536) and DLL4 (ab7280) were purchased from Abcam (Cambridge, UK. Monoclonal antibody to β-actin and 4, 6-diamidino-2-phenylindole (DAPI) were purchased from Sigma (St Louis, MO, USA). The chemical inhibitors including Gamma secretase inhibitor (565770), STAT3 inhibitor (573097) and the JAK inhibitor (420099) were bought from Calbiochem (San Diego, CA, USA). Recombinant human CXCL8/IL-8 protein was purchased from R & D systems and recombinant protein IL-6 was bought from EMD Millipore.

### Cell culture

The human breast epithelial cell line MCF7, SKBR3 and the mouse breast epithelial cell line 4T1 were purchased from the American Type Culture Collection (Manassas, VA). All cell cultures were maintained in a humidified 5% CO_2_ atmosphere at 37°C. The hormone receptor status of the MCF7 cells is estrogen receptor positive (ER+), progesterone positive (PR+) and HER2 negative (HER2-) while that of SKBR3 is ER- PR- HER2+. MCF7, SKBR3 and 4T1 cells were grown in the minimum Eagle's medium, RPMI medium and DMEM medium respectively. All of these media were supplemented with 10% fetal bovine serum, penicillin (100 units/ml), and streptomycin (100 μg/ml). All cell culture products were purchased from GIBCO (Seoul, Korea). The gamma-secretase inhibitor (GSI) was used in cancer cells at a concentration of 20 μM; the JAK inhibitor was used at 10 μM, and the STAT3 inhibitor was used at 2 μM.

### Irradiation

Cancer cells were exposed to radiation using a 137Cs γ-ray source (Atomic Energy of Canada, Ltd, Mississauga, Canada) at a dose rate of 3.81 Gy/min. Further analysis such as migration, invasion assay, western blot and immunocytochemical analysis were done at 48 hours after fractionated irradiation (2 Gy × 3; 2 Gy per day for 3 days).

### Transfection

Cells were transfected with siRNA duplexes (40 nM) by using Lipofectamine 2000 (Invitrogen), following the procedure recommended by the manufacturer. Irradiation was performed 48 hr after transfection. All siRNA were purchased from Genolution Pharmaceuticals, Inc (Seoul, Korea).

### Boyden chamber invasion and migration assays

Matrigel invasion assays were conducted using 24-well Transwell inserts (Corning Inc., Tewksbury, MA, USA) pre-coated with 10 mg/ml growth-factor-reduced Matrigel (BD Biosciences) at 37 °C. All breast cancer cells (2 × 10^4^) suspended in 200 μl of serum-free medium were seeded into the upper chamber with the lower well filled with 0.8 ml of a growth medium. After incubation for 48 hr (hours), migrated cells were fixed and stained with a Diff-Quick kit (Fisher) and photographed and invasiveness was calculated as described in our previous report [36]. A migration assay was also performed in Transwell inserts containing similar membranes without the Matrigel coating.

### Western blot analysis

Cell lysates were prepared by incubation with a lysis buffer (40 mM Tris-HCl pH 8.0, 120 mM NaCl, 0.1% Nonidet-P40) supplemented with protease inhibitors. Then proteins were then subjected to SDS–polyacrylamide gel electrophoresis (SDS-PAGE) before being transferred to a nitrocellulose membrane (Amersham, Arlington Heights, IL). Primary antibodies were used to detect the relevant protein, and β-actin was used as loading control. Blots were developed with horseradish peroxidase (HRP)-conjugated secondary antibodies and proteins were visualized using enhanced chemiluminescence (ECL) (Amersham, Arlington Heights, IL), according to the manufacturer's protocol. Secondary antibodies, anti-mouse IgG-HRP, anti-goat IgG-HRP and anti-rabbit IgG-HRP were purchased from Santa Cruz biotechnology (CA, USA).

### Clonogenic survival assay

MCF7 and SKBR3 cells were seeded into 60 mm cell culture dish at concentrations of 300–400 cells per plate after the knockdown of si-Notch2 and radiation afterwards with dose rate of 1 Gy-4 Gy. The cells were incubated for 8–10 days to allow for colony formation. The colonies composed of more than 50 cells were counted. Survival fraction was calculated as (mean colonies counted. Each assay was performed in triplicates.

### Immunohistochemistry

Athymic balb/c nude mice were euthanized and tissues were harvested and fixed in formalin for the preparation of paraffin sections. Paraffin-embedded tissue sections were deparaffinized in xylene, 95, 90, and 70% ethanol, followed by phosphate buffered saline (PBS). Epitopes were unmasked with 20 mg/ml proteinase K in PBS with 0.1% Triton X-100. Sections were stained with hematoxylin and eosin (H & E) or immunostained overnight at 4°C with the Notch2 (1:100), E-cadherin (1:100), N-cadherin (1:100), vimentin antibody (1:100) and IL-6 antibody (1:100). After washing in PBS, a 1:200 dilution of biotinylated goat anti-rabbit IgG or anti-mouse IgG antibody in a blocking solution was applied to the sections and incubated for 30 min. After washing in PBS, the ABC reagent (ABC Peroxidase Standard Staining Kit-Thermo Fisher Scientific) was applied to the sections and incubated for 30 min. After washing in PBS, color reaction was performed with 3, 30-diaminobenzidine (Vector Laboratories) and the slides were washed with PBS. After counter-staining with hematoxylin and clearing with a graded ethanol series and xylene, the sections were mounted with Canada balsam. Observations and photography were conducted using a microscope (Olympus) equipped with a DP71 digital imaging system (Olympus).

### Immunocytochemistry

Cells were fixed with 4% paraformaldehyde and permeabilized with 0.1% Triton X-100 in phosphate-buffered saline (PBS). Following fixation, the cells were incubated at 4°C overnight with mouse polyclonal anti-human E-cadherin (1:200) N-cadherin (1:200), anti-Jagged1 (1:200), DLL4 (1:200), Rabbit polyclonal anti-human anti-vimentin (1:200), and goat polyclonal anti-human anti-SLUG (1:200) primary antibody in PBS with 1% bovine serum albumin and 0.1% Triton X-100. Immunostaining of the proteins was visualized using Alexa Fluor 488-conjugated anti-rabbit and anti-mouse or anti-goat secondary antibodies (Molecular Probes, Seoul, Korea). Nuclei were counterstained with DAPI (Sigma-aldrich). Immunostaining was observed with an Olympus IX71 fluorescence microscope (Olympus, Seoul, Korea).

### Real time quantitative PCR analysis

Total cell RNA was isolated using the Trizol reagent (Invitrogen). RT–PCR was performed with Super-Script III (Invitrogen) according to the manufacturer's instructions. A real time qPCR analysis was set up with the KAPA SYBR FAST qPCR Master Mix (2X) and carried out in a Rotor Gene Q system (Qiagen, Korea). All primers used in this study were purchased from Macrogen, Korea.

### Animal experiments

Green fluorescence protein (GFP)-labeled 4T1 breast cancer (1 × 10^6^) cells were injected into the fourth mammary fat pad of athymic Balb/c female nude mice (5 weeks of age; Orient). Irradiation (2 Gy × 5; 2 Gy/day for 5 days) was then done five times every day. Tumor sizes were measured with a caliper (calculated volume = shortest diameter^2^ × longest diameter/2) at three day intervals. This study was reviewed and approved by the Institutional Animal Care and Use Committee (IACUC) of the Center for Laboratory Animal Sciences, the Medical Research Coordinating Center, and the HYU industry-University Cooperation Foundation.

### Statistical analysis

All experimental data are reported as means; error bars represent the standard deviation (SD) of at least three independent tests. Statistical analyses were performed using non-parametric Student's *t*-tests to assess the significance levels. Results were considered as significant if *p < 0.05, **p < 0.01, ***p < 0.001.

## SUPPLEMENTARY MATERIALS FIGURES


