# Supplementary material for: Radiation driven epithelial-mesenchymal transition is mediated by Notch signaling in breast cancer
Source: Oncotarget. 2016 Jul 23;7(33):53430–42. doi: 10.18632/oncotarget.10802 (PMC5288197; doi:10.18632/oncotarget.10802)
Supplement: Supplementary file 1 [file oncotarget-07-53430-s001.pdf]

Radiation driven epithelial-mesenchymal transition is mediated by Notch signaling in breast cancer

Supplementary Materials

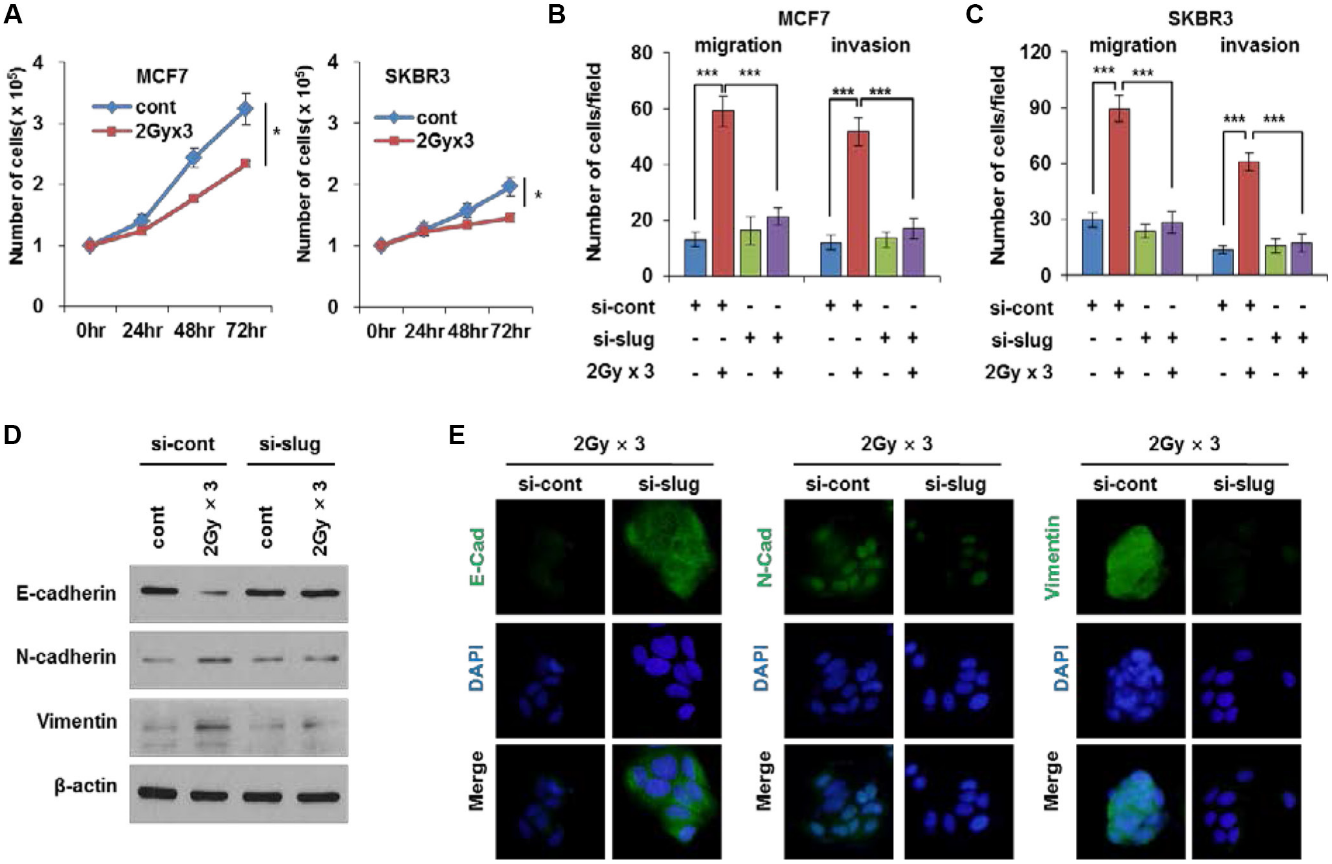

Supplementary Figure S1: Fractionated radiation promotes EMT of breast cancer cells through SLUG. (**A**) Cell growth of MCF7 and SKBR3 cells after fractionated irradiation (2Gy × 3; 2 Gy/day for 3 days). (**B** and **C**) Migration and invasion assay in transwells after fractionated irradiation of MCF7 and SKBR3 cells, respectively, that are transfected with siRNA targeting slug. (**D**) Western blot for EMT markers such as E-cadherin, N-cadherin and vimentin in irradiated MCF7 breast cancer cells that are transfected with siRNA targeting slug. (**E**) Immunocytochemistry for EMT markers such as E-cadherin, N-cadherin and vimentin in irradiated MCF7 cells transfected with siRNA targeting slug. β-actin was used as a loading control. Error bars represent mean ± S.D. of triplicate samples. *$p < 0.05$.

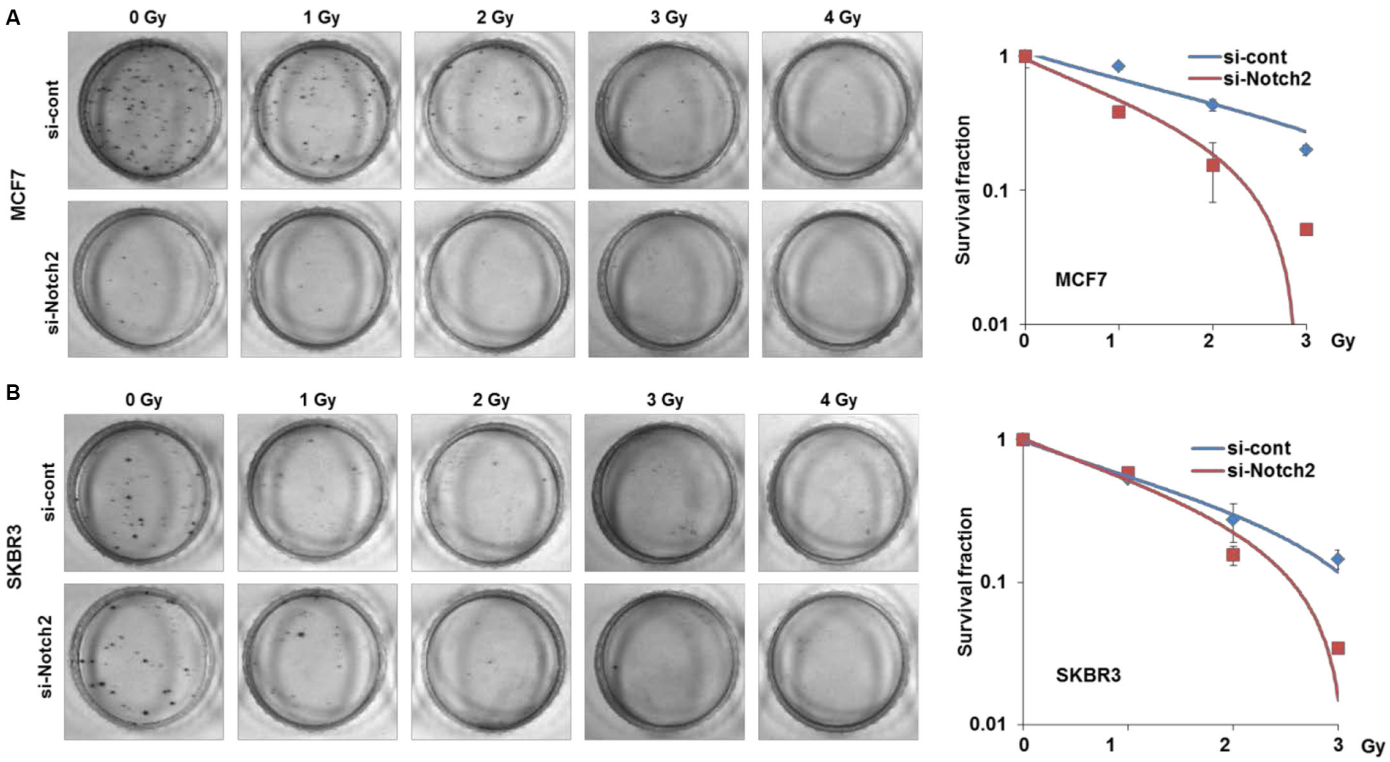

Supplementary Figure S2: Fractionated radiation-induced Notch2 signaling decreases cell clonogenicity in breast cancer cells. (**A**) Clonogenic assay of irradiated (0–4 Gy) MCF7 cells that are transfected with siRNA targeting Notch2. (**B**) Clonogenic assay of irradiated (0–4 Gy) SKBR3 cells that are transfected with siRNA targeting Notch2.

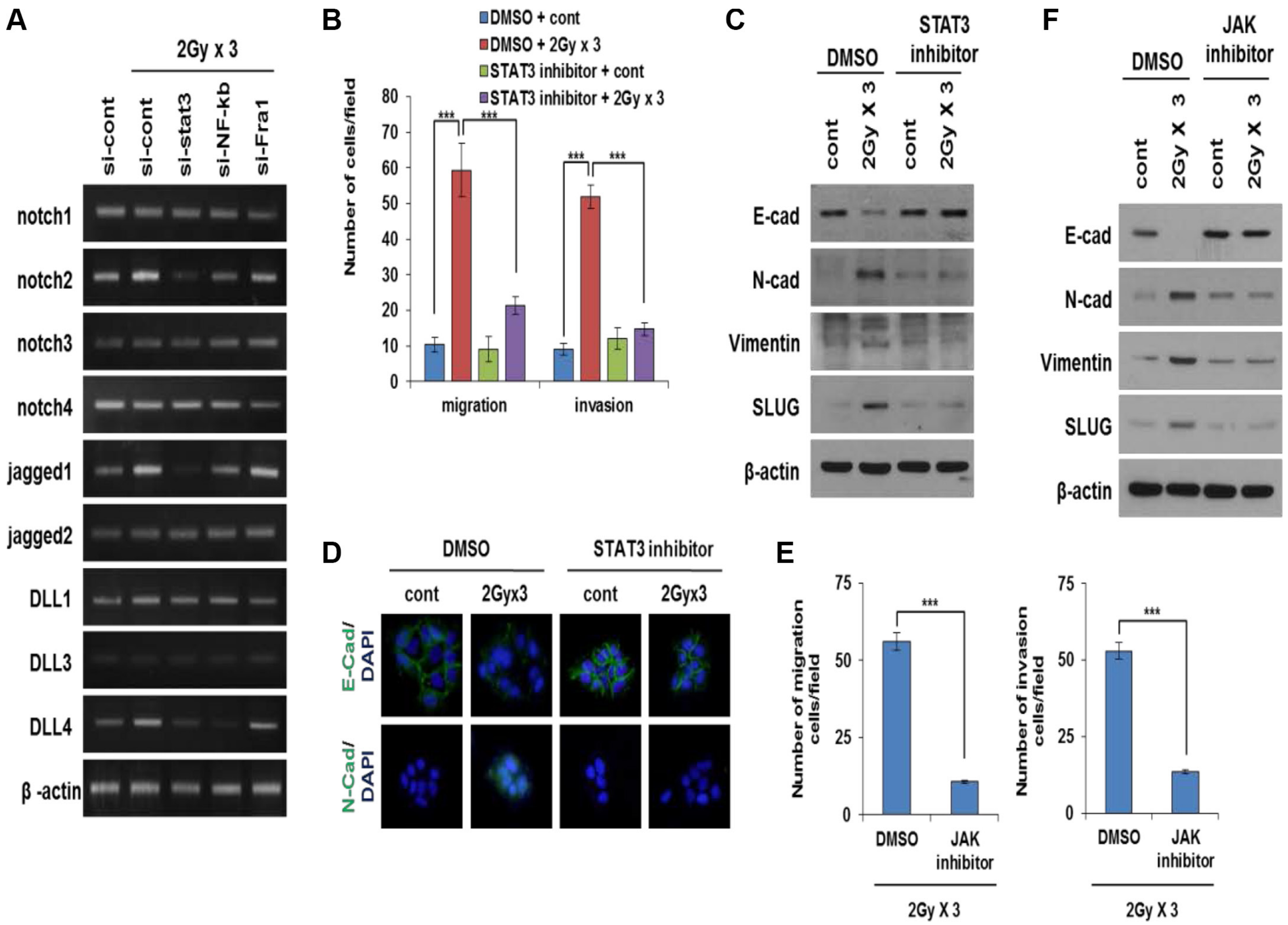

Supplementary Figure S3: Fractionated radiation-induced JAK/STAT signaling promotes EMT in breast cancer cells. (**A**) PCR analysis for notch family in fractionation-radiated (2 Gy × 3) MCF7 cells that are transfected with siRNA targeting STAT3, NF-κB and Fra-1. (**B**) Migration and invasion assay in transwells after irradiation of MCF7 that are treated with STAT3 inhibitor (2 μM). (**C**) Western blot for EMT markers after fractionated irradiation of MCF7 that are treated with STAT3 inhibitor. (**D**) Immunocytochemistry for EMT markers, E-cadherin in irradiated MCF7 cells that are treated with STAT3 inhibitor (2 μM). (**E**) Migration and invasion assay in transwells after irradiation of MCF7 that are treated with JAK inhibitor (10 μM). (**F**) Western blot for EMT markers after fractionated irradiation of MCF7 that are treated with JAK inhibitor. β-actin was used as a loading control. Error bars represent mean ± S.D. of triplicate samples. ***$p < 0.001$.

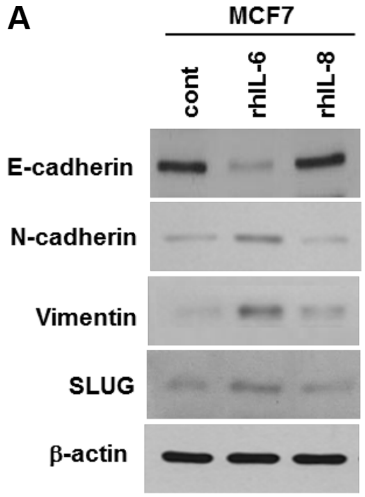

Supplementary Figure S4: IL-6 regulates the EMT more efficiently than IL-8 in breast cancer cells by fractionated radiation. (**A**)) Western blot for EMT markers after recombinant IL-6 (0.5 ng/mL) and recombinant IL-8 (2 μg/mL) protein in MCF7 cells. β-actin was used as a loading control. Error bars represent mean ± S.D. of triplicate samples. **$p < 0.01$, ***$p < 0.001$.

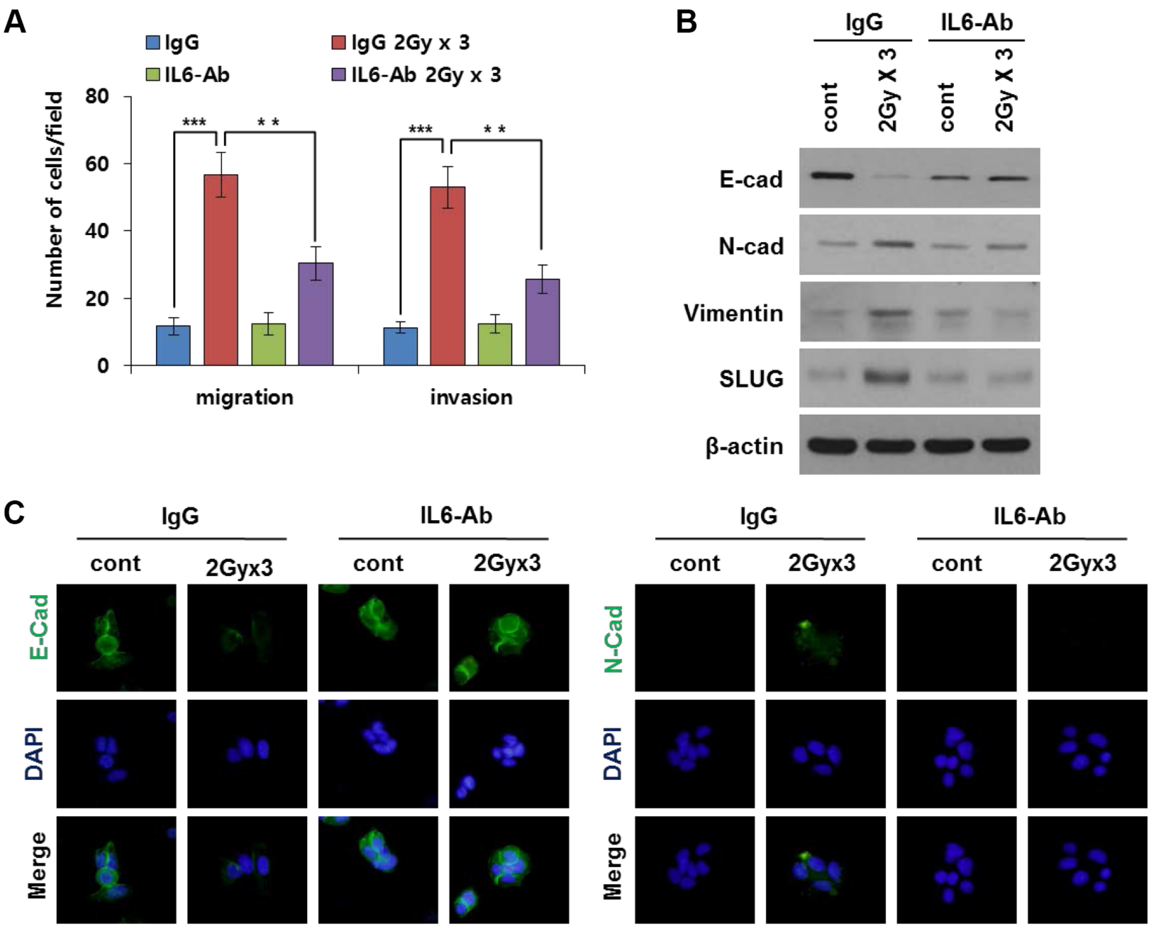

Supplementary Figure S5: IL-6 regulation the EMT in breast cancer cells by fractionated radiation. (**A**) Migration and invasion assay in transwells after irradiation of MCF7 that are treated with IL-6 neutralizing antibody. (**B**) Western blot and (**C**) immunocytochemistry for EMT markers after fractionated irradiation of MCF7 that are treated with STAT3 inhibitor. β-actin was used as a loading control. Error bars represent mean ± S.D. of triplicate samples. $^{**}p < 0.01$, $^{***}p < 0.001$.

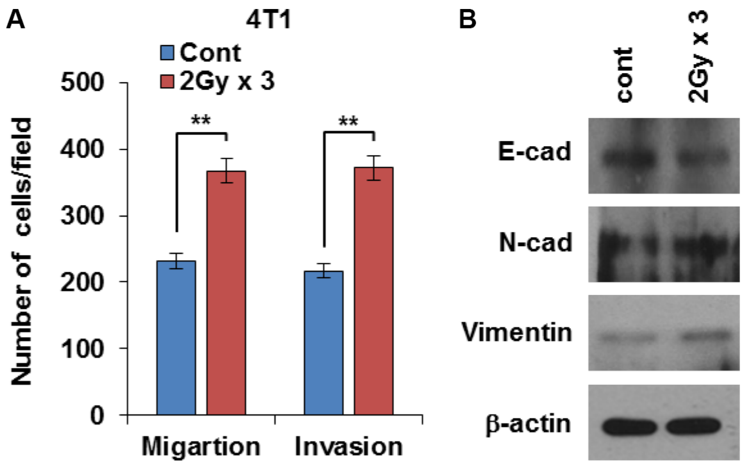

Supplementary Figure S6: Fractionated radiation induces invasiveness in 4T1 mouse mammary cells. (**A**) Migration and invasion assay in transwells after irradiation of 4T1. (**B**) Western blot for EMT markers after fractionated irradiation of 4T1 cells. β-actin was used as a loading control. Error bars represent mean ± S.D. of triplicate samples. $^{**}p < 0.01$.
